# Effect of Urinary Incontinence on the Quality of Life of Older Adults in Riyadh: Medical and Sociocultural Perspectives

**DOI:** 10.7759/cureus.11599

**Published:** 2020-11-20

**Authors:** Sulaiman Alshammari, Malak A Alyahya, Reema S Allhidan, Ghadeer A Assiry, Hissah R AlMuzini, Munirah A AlSalman

**Affiliations:** 1 Family and Community Medicine, College of Medicine, King Saud University, Riyadh, SAU

**Keywords:** urinary incontinence, quality of life, older adults

## Abstract

Objectives

The objective of the study is to determine the impact of urinary incontinence (UI) on the quality of life (QoL) of the Saudi elderly population. Besides, we investigated individuals’ help-seeking behaviors and the religious and cultural aspects of UI among the Saudi elderly.

Methods

We conducted a cross-sectional study using a random sample of 150 Saudi older adults of both genders. The participants were outpatient clinics of government and private hospitals in Riyadh from January-March 2019. Volunteer medical students interviewed and distributed a standard questionnaire form (Arabic version of the ICIQ-SF [International Consultation on Incontinence Questionnaire] with 33 additional questions, four of which came from them from the King's Health Questionnaire (KHQ) to the targeted population.

Results

In this study, there were 124 elderly participants (response rate 83%), of whom 62.9% were women. The mean age was 71.9 (±7.8). The "moderate" and "severe" ICIQ scores account for 78 (62.9%) and 32 (25.8%), respectively. There was a significant association between the ICIQ scores severity of UI and increasing BMI, nocturnal diuresis, urinary tract infection, and lung diseases. However, 36.3% of participants did not seek help due to misconceptions about UI and aging, unavailability of treatment, and embarrassment of sharing such symptoms with others. The participants suffered from limitations of social life (36.3%), a negative impact on their physical activity (18.5%), personal hygiene (21.8%), and their self-esteem (32.3%). About 17% and 33.1% of participants repeat ablution and prayers of participants, respectively.

Conclusions

Urinary incontinence (UI) is a common and distressing problem in the elderly. A large proportion of the participants had a detrimental effect on their quality of life. A substantial percentage of older adults did not seek help. As a result, we recommended raising awareness about UI, QoL, the misconceptions, and encourage them to overcome the stigma of embarrassment and seek medical help.

## Introduction

Urinary incontinence (UI) is a common problem among older adults worldwide. The prevalence among older women and men varies from 17% to 55% and from 11% to 34%, respectively [1]. The International Continence Society defined UI as the occurrence of any complaints of involuntary urine leakage, which is considered a social or hygienic problem [2]. A previous Saudi national household study found that 6.4-9.0% of older adults suffered from UI [3]. The prevalence of UI was 55.2% among Malaysian women [4], 50% for Egyptian women [5]. However, the prevalence in five Western countries, including Canada, Germany, Italy, Sweden, and the United Kingdom, was between 7.5 and 29.7% [3,6]. Previous studies showed that the risk factors of UI included an age of ≥ 35, low socioeconomic status, multiparty, neonatal macrosomia, and prolonged second stage of labor [7].

The evidence from a Danish study indicated a strong association between first vaginal delivery and UI one year after the delivery [8]. However, UI is usually an under-reported problem, as the vast majority of older adults share a common belief that UI is not manageable. Other individuals assume that it is a normal physiological part of aging. Consequently, they neglect it or just hesitate to report symptoms due to the culturally embarrassing and sensitive nature of the issue [4]. There is no doubt that the inability to control urine is an unpleasant feeling and a distressing problem. Although it does not lead to death, it can affect an individual’s quality of life significantly [9]. The reported potential physical and psychosocial burdens affect the patients and their caregivers [10,4]. 

A study of urinary incontinence among Moroccan and Turkish migrant women found that UI constituted a significant problem in their hygiene and purifying routines [11]. As Muslims, they have to perform prayers that are preceded by ablution five times per day, and the urinary incontinence breached their status of purity. As a result, they had to wash more often and experienced this as a heavy burden. Despite its high prevalence among older adults worldwide, there are very few studies investigating QoL in older adults with UI; most of them have investigated UI among women only. Also, there is little awareness of its effect on QoL among Saudi older adults.

Understanding the current prevalence of UI will draw the attention of the community to this issue. As a result, we aimed to determine the effect of UI on their QoL. In addition, we investigated individuals’ help-seeking behaviors and the religious and cultural aspects of UI among the Saudi elderly.

## Materials and methods

We conducted a cross-sectional study using a random sampling technique for 150 older adults in Riyadh from January-March 2019. We estimated the sample size using the formula for single-proportion sample size as follows: n = Z2α P(1-P)/d2 Zα = ه1.96 for 95% confidence level, d = precision is 0.05, and P = 0.11, which was the percentage of elderly individuals who were at least 60 years of age who had UI based on the definition reported in the literature [1]. So n = (1.96)² (0.11) (1-0.11) / (0.05)² = 150. Participants were Saudi men and women who were at least 60 years of age and attended the outpatient clinics of government and private hospitals in Riyadh, Saudi Arabia. The authors excluded participants if they had gynecological or lower urinary tract surgery in the past three months or declined to participate. Trained volunteer medical students interviewed participants and distributed a standard questionnaire form (Arabic version of the International Consultation on Incontinence Questionnaire (ICIQ)-Urinary Incontinence Short Form [12,13]. We added eleven additional questions about demographics, nine questions about risk factors, four questions regarding social/physical limitations from the King’s Health Questionnaire [14], six questions regarding cultural aspects, and three questions about help-seeking behaviors to the target population. We measured the height and weight of participants to calculate their body mass index (BMI). We used the following definitions of UI. Urge UI is a sudden and intense urge to urinate, followed by an involuntary loss of urine. Stress UI is urine leaks when a person exerts pressure on their bladder by coughing, sneezing, laughing, exercising, or lifting heavy objects. Nocturnal UI is involuntary urination while asleep after the usual age at which bladder control begins. Physical activity-induced UI is urinary incontinence associated with exercise. Overflow UI is frequent or constant dribbling of urine due to a bladder that does not empty, while idiopathic UI is of unknown cause. The ICIQ-UI Short Form is a questionnaire for evaluating the frequency, severity, and impact on the quality of life (QoL) of urinary incontinence in men and women in research and clinical practice across the world. This short and straightforward questionnaire is also of use to general practitioners and clinicians in both primary and secondary care institutions to screen for incontinence and obtain a brief yet comprehensive summary of the level, impact, and perceived cause of incontinence symptoms to facilitate patient-clinician discussions. The questionnaire consists of 4 items: frequency of urinary incontinence, amount of leakage, overall impact of urinary incontinence, and self-diagnostic item. Its validity, reliability, and responsiveness have been established in many languages, including Arabic Scoring scale: 0-21 [15,16].

Five academic experts (three family physicians, a preventive medicine professor, and a urologist) reviewed the questionnaire. We piloted the Arabic self-administered questionnaire on twenty participants to ascertain that the questions were understood, to estimate the time required to fill in the questionnaire, and to explore the obstacles and constraints. At the same time, the investigators revised the questionnaire and tested its clarity and feasibility. People who participated in the pilot study spent between 10-15 minutes to complete the questionnaire. We excluded them from the main study. We analyzed the data using the Statistical Package for Social Studies (SPSS 22; IBM Corp., Armonk, NY, USA). The investigators used frequencies, percentages, chi-squared tests, and P-values ≤ 0.05 to report statistical significance. 

## Results

Of 150 eligible participants, 124 took part in this study (response rate 83%), of which 62.9% were women. More than 50% of the participants were aged seventy years and above. About a third of the participants were illiterate, more of these were women. Additionally, 70% percent were married, and 6.5% were currently working. Table 1 shows the distribution of socio-demographic characteristics of participants. 

**Table 1:** Socio-demographic characteristics of the participants, N=124 (100%).

Variables	124 (100%)	Male 46 (37.1)	Female 78 (62.9)	p-value
Age (years)				
60-69	53 (42.7)	16 (34.8)	37 (47.4)	
70-79	45 (36.3)	17 (37)	28 (35.9)	
≥80	26 (21.0)	13 (28.3)	13 (16.7)	0.22
Educational level				
Illiterate	40 (32.3)	5 (10.9)	35 (44.9)	
Below high school	45 (36.3)	20(43.5)	25(32.1)	
Above high school	39 (31.5)	21(45.7)	18(23.1)	0
Marital status				
Unmarried	37 (29.8)	7(15.2)	30(38.5)	
Married	87 (70.2)	39 (84.8)	48 (61.5)	0.006
Occupational status				
Current working	8 (6.5)	5 (10.9)	3 (3.8)	
Not working	116 (93.5)	41 (89.1)	75 (96.2)	0.1
Monthly income				
< 5000	49 (39.5)	8 (17.4)	41 (52.6)	
5000-10000	52 (41.9)	26 (56.5)	26 (33.3)	
> 10000	23 (18.5)	12 (26.1)	11 (14.1)	0.001

Concerning the participant’s gender in the context of BMI, smoking, the number of children, and the age of menopause is illustrated in Table 2.

**Table 2:** Participants' gender in the context of BMI, smoking, number of children and the age of menopause, N=124 (100%).

Variables	124 (100%)	Male 46 (37.1)	Female 78 (62.9)	P-value
BMI				
Normal weight	26 (21.0)	13 (28.3)	13 (16.7)	
Overweight	67 (54.0)	25 (54.3)	42 (53.8)	
Obese	31 (25.0)	8 (17.4)	23 (29.5)	0.1
Smoking or Shisha use				
Yes	12 (9.7)	8 (17.4)	4 (5.1)	
No	112 (90.3)	38 (82.6)	74 (94.9)	0.02
Number of children				
0-4	18 (23.1)		18 (23.1)	
≥5	60 (76.9)		60 (76.9)	
No. vaginal delivery				
0-4	25 (32.1)		25(32.1)	
≥5	53(67.9)		53(67.9)	
Age at menopause				
≤50	61(78.2)		61(78.2)	
≥50	17(21.8)		17(21.8)	

**Figure 1:**
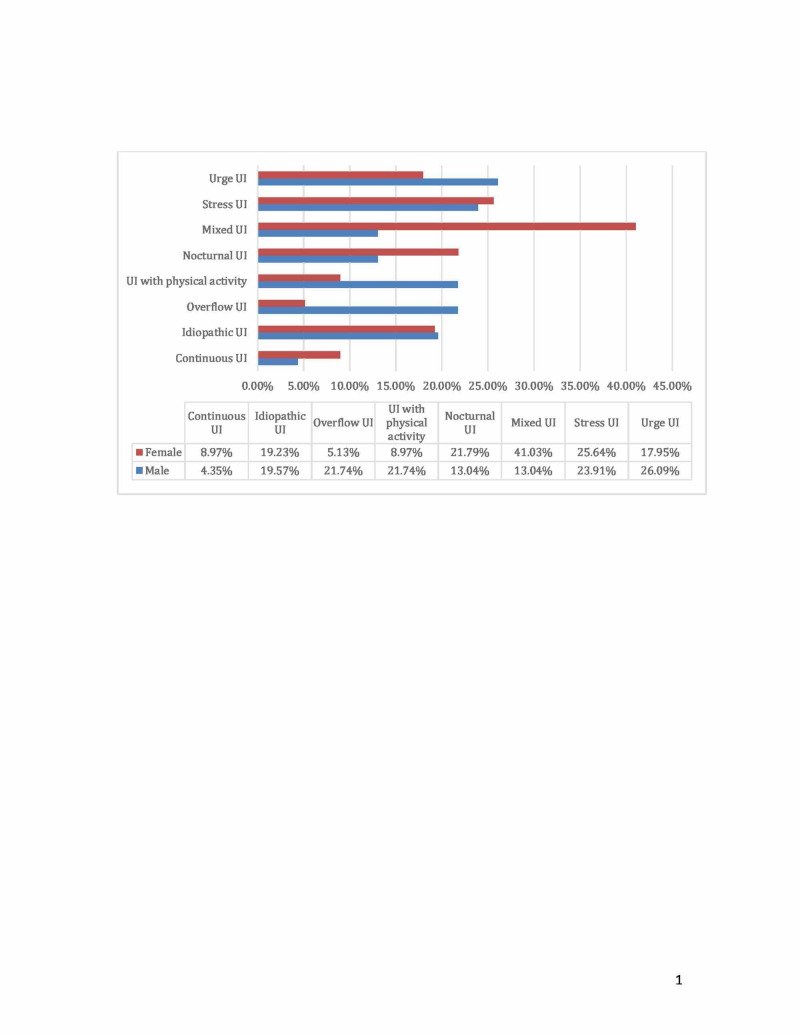
The types of urinary incontinence by sex of the older adults in Riyadh.

Overweight and obesity affected 79.0% of the participants, and about 10% were smokers with about triple the smoking rate in men than women. About 80% had five children, and more than about 70% of them were the product of vaginal delivery. Menopause took place at 50 years of age and above in 21.9% of the participants. Overall, the percentages of UI is urge UI 26 (20.9)%, stress UI 31 (25.0%), nocturnal UI 23 (18.5%), physical activity-induced UI 17 (13.7%), overflow UI 14 (11.3%), and idiopathic UI 24 (19.4%). Of the 78 women, the most prevalent types of UI were as follows: mixed UI 32 (41.0%), stress UI 20 (25.6%), and nocturnal UI 17 (21.7%). Of the 46 men, the most prevalent types of UI were as follows: urge UI 12 (26.1%), stress UI 11 (23.9%), overflow UI 10 (21.7%), and UI associated with physical activity 10 (21.7%). Figure 1 illustrated the types of UI by sex of older adults. 

We found that the ICIQ scores among our participants were Mild 14 (11.3%), Moderate 78 (62.9%), and Severe 32 (25.8%). There was a significant association between increasing BMI and the ICIQ scores severity of UI, showing that of the 124 older Saudi adults enrolled, 67 (54.0) were overweight (p< 0.05). The majority of participants who had moderate 47 (37.9%) or severe 16 (12.9%) UI were obese. Other factors such as age, gender, number of vaginal deliveries, and menopause age did not show statistically significant differences in the ICIQ scores severity of UI (Table3). 

**Table 3:** Associated risk factors versus the severity of urinary incontinence.

Risk factors	Severity according to ICIQ score N (%)				p-value
	Mild	Moderate	Severe	Total	
	14 (11.3%)	78 (62.9%)	32 (25.8%)	124 (100%)	
Gender: Male	5 (10.9) [35.7]	30 (65.2) [38.5]	11 (23.9) [34.4]	46 (100) [37.1]	0.9
Female	9 (11.5) [64.3]	48 (61.5) [61.5]	21 (26.9) [65.6]	78 (100) [62.9]	
Age 60-69	8 (15.1) [57.1]	36 (67.9) [46.2]	9 (17) [28.1]	53 (100) [42.7]	0.2
70-79	5 (11.1) [35.7]	25 (55.6) [32.1]	15 (33.3) [46.9]	45 (100) [36.3]	
80-90	1 (3.8) [7.1]	17 (65.4) [21.8]	8 (30.8) [25.0]	26 (100) [21.0]	
BMI Normal weight	6 (23.1) [42.9]	16 (61.5) [20.5]	4 (15.4) [12.5]	26 (100.0) [21.0]	0.04
Overweight	4 (6.0) [28.6]	47 (70.1) [60.3]	16 (23.9) [50.0]	67 (100.0) [54.0]	
Obese	4 (12.9) [28.6]	15 (48.4) [19.2]	12 (38.7) [37.5]	31 (100.0) [25.0]	
No.vaginal deliveries					0.4
0-4	2(8.0)[22.2]	18(72.0) [37.5]	5(20.0) [23.8]	25 (100.0) [32.1]	
≥5	7 (13.2) [77.8]	30 (56.6) [65.2]	16 (30.2) [76.2]	53 (100.0) [67.9]	
Age at menopause					0.6
<50	6(9.8) [66.7]	38(62.3) [79.2]	17(27.9) [80.9]	61 (100.0) [78.2]	
≥50	3(17.6) [33.3]	10(58.8) [20.8]	4 (37.5) [19.1]	17 (100.0) [21.8]	

Our study showed the associated health conditions (risk factors) versus the severity of urinary incontinence ICIQ score. The percentage of those who have never had UTI (37.9 %), rarely have UTI (24.2%), and often have UTI (37.9%) suffered from UI. Overall, 59.7%, 32.3%, 37.0%, 16.1%, 21%, 18.5%, and 4.8% of those with UI suffered from nocturnal diuresis, arthritis, benign prostatic hypertrophy, fecal incontinence, heart disease, stroke, and none of the above conditions, respectively.

The majority of those with UI also had hypertension (56.5%), diabetes (79.0%), and nocturnal diuresis (59.7%). Most of the participants with UI had no lung diseases 116 (93.5%). Most of the participants with nocturnal diuresis 71 (95.9%) suffer moderate to severe ICIQ scores. Upon investigating the association between urinary tract infections (UTIs) and UI severity, most of those with UI had experienced UTIs at some point in their lifetime 77 (62.1%). The majority of the participants who had UTIs 74 (96.1%) suffered moderate to severity ICIQ scores. Fecal incontinence, hypertension, diabetes mellitus, heart diseases, arthritis, stroke, and benign prostatic hypertrophy did not significantly impact the ICIQ score (Table 4). 

**Table 4:** Associated health conditions (risk factors) versus the severity of urinary incontinence. N.B: ( ) is for horizontal percentage, whereas square bracket is for vertical percentage. ICIQ=International Consultation on Incontinence Questionnaire; BMI=body mass index; BPH=benign prostatic hypertrophy

Associated risk factors	Severity according to ICIQ score N (%)				p-value	
	Mild	Moderate	Severe	Total		
Fecal incontinence Yes	1 (5.0) [7.1]	12 (60.0) [15.4]	7 (35.0) [21.9]	20 (100) [16.1]	0.439	
No	13 (12.5) [92.9]	66 (63.5) [84.6]	25 (24.0) [78.1]	104(100.0) [83.9]		
Nocturnal diuresis Yes	3 (4.1) [21.4]	48 (64.9) [61.5]	23 (31.1) [71.9]	74 (100.0) [59.7]	0.005	
No	11 (22.0) [78.6]	30 (60.0) [38.5]	9 (18.0) [28.1]	50 (100.0) [40.3]		
Urinary tract infection					0.007	
Never	11 (23.4) [78.6]	24 (51.1) [30.8]	12 (25.5) [37.5]	47 (100.0) [37.9]		
Rarely	3(10.0) [21.4]	18 (60.0) [23.1]	9 (30.0) [28.1]	30 (100.0) [24.2]		
Often or Always	0 (0.0) [0.0]	36 (76.6) [46.2]	11 (23.4) [34.4]	47 (100.0) [37.9]		
Hypertension Yes	9 (12.9) [64.3]	42 (60.0) [53.8]	19 (27.1) [59.4]	70 (100.0) [56.5]	0.713	
No	5 (9.3) [35.7]	36 (66.7) [46.2]	13 (24.1) [40.6]	54 (100.0) [43.5]		
Diabetes mellitus Yes	9 (9.2) [64.3]	62 (63.3) [79.5]	27 (27.6) [84.4]	98 (100.0) [79.0]	0.301	
No	5 (19.2) [35.7]	16 (61.5) [20.5]	5 (19.2) [15.6]	26 (100.0) [21.0]		
Lung diseases Yes	1 (12.5) [7.1]	2 (25.0) [2.6]	5 (62.5) [15.6]	8 (100.0) [6.5]	0.04	
No	13 (11.2) [92.9]	76 (65.5) [97.4]	27 (23.3) [84.4]	116 (100.0) [93.5]		
Heart diseases Yes	0 (0.0) [0.0]	17 (65.4) [21.8]	9 (34.6) [28.1]	26 (100.0) [21.0]	0.094	
No	14(14.3) [100.0]	61 (62.2) [78.2]	23 (23.5) [71.9]	98 (100.0) [79.0]		
Arthritis Yes	3 (7.5) [21.4]	25 (62.5) [32.1]	12 (30.0) [37.5]	40 (100.0) [32.3]	0.561	
No	11 (13.1) [78.6]	53 (63.1) [67.9]	20 (23.8) [62.5]	84 (100.0) [67.7]		
Stroke Yes	1 (4.3) [7.1]	14 (60.9) [17.9]	8 (34.8) [25.0]	23 (100.0) [18.5]	0.349	
No	13 (12.9) [92.9]	64 (63.4)[82.1]	24 (23.8) [75.0]	101 (100.0) [81.5]		
BPH Yes	0 (0.0) [0.0]	12 (70.6) [40.0]	5 (29.4) [45.5]	17 (100.0) [37.0]	0.183	
No	5 (17.2) [100.0]	18 (62.1) [60.0]	6 (20.7) [54.5]	29 (100.0) [63.0]		
None Yes	0 (0.0) [0.0]	3 (50.0) [3.8]	3 (50.0) [9.4]	6 (100.0) [4.8]	0.315	
No	14(11.9) [100.0]	75 (63.6) [96.2]	29 (24.6) [90.6]	118 (100.0) [95.2]		

In our study, 36.3% of participants did not seek help from anybody. Those participants suffering from moderate to severe ICIQ scores sought help from others rather than their counterparts, p<0001. Those who sought help or shared their problems with doctors, pharmacists, friends, and family members account for 9.7%, 8.1%, 8.1%, and 5.6%, respectively. There was no statistically significant association between seeking help from different categories of potential helpers and the UI’s severity based on the ICIQ score (Table 5).

**Table 5:** Association between help-seeking behaviors and severity according to ICIQ score. N.B: Round brackets ( ) are for horizontal variables, whereas square brackets [] are for vertical variables. Abbreviations: ICIQ=International Consultation on Incontinence Questionnaire

	Severity according to ICIQ score N (%)				
				Source of help-seeking	Mild	Moderate	Severe	Total	p-value
Yes	1 (1.3)[7.1]	56 (70.9)[71.8]	22 (27.8)[68.8]	79 (100.0) [63.7]	0
None	13 (28.9)[92.9]	22 (48.9)[28.2]	10 (22.2)[31.3]	45 (100.0)[36.3]	
Doctors Yes	0 (0.0)[0.0]	8 (66.7)[10.3]	4 (33.3)[12.5]	12 (100.0)[9.7]	0.402
No	14 (12.5)[100.0]	70 (62.5)[89.7]	28 (25.0)[87.5]	112 (100.0)[90.3]	
Pharmacist Yes	1 (10.0 )[7.1]	8 (80.0)[10.3]	1 (10.0)[3.1]	10 (100.0)[8.1]	0.455
No	13 (11.4)[92.9]	70 (61.4)[89.7]	31 (27.2)[96.9]	114 (100.0)[91.9]	
Friends Yes	0 (0.0)[0.0]	7 (70.0)[9.0]	3 (30.0)[9.4]	10 (100.0)[8.1]	0.499
No	14 (12.3)[100.0]	71 (62.3)[91.0]	29 (25.4)[90.6]	114 (100.0)[91.9]	
Family Yes	0 (0.0)[0.0]	6 (85.7)[7.7]	1 (14.3)[3.1]	7 (100.0)[5.6]	0.4
No	14 (12.0)[100.0]	72 (61.5)[92.3]	31 (26.5)[96.9]	117 (100.0)[94.4]	

The urinary incontinence participants suffered from limitations of social life (36.3%), a negative impact on their physical activity (18.5%), and personal hygiene (21.8%). Additionally, some participants noticed a negative impact on their ability to travel (26.6%) or visiting others or being visited by them (37.9%), and their self-esteem (32.3%). Repeat ablution and prayers were reported by 16.9% and 33.1% of participants, respectively. However, there were no significant statistical differences between men and women, as shown in Table 6.

**Table 6:** Effect of urinary incontinence on participants by gender.

Effect of urinary incontinence on participant				
Physical and psychosocial conditions	Total	Gender		p-value
		Male N(%)	Female N(%)	
Impact on physical activities	23(18.5)	8(17.4)	15(19.2)	0.7
Travel ability	33(26.6)	13(28.3)	20(25.6)	0.7
Social life limitations	45(36.3)	16(34.8)	29(37.2)	0.7
Visiting ability	47(37.9)	17(37.0)	30(38.5)	0,8
Repeat ablution	21(16.9)	9(19.6)	12(15.4)	0.5
Repeat prayers	41(33.1)	14(30.4)	27(34.6)	0.6
Feel nervous or anxious	29(23.4)	9(19.6)	20(25.6)	0.4
Impact on personal hygiene	27(21.8)	10(21.7)	17(21.8)	0.9
Low self-esteem	40(32.3)	14(30.4)	26(33.3)	0.7

The reasons for not seeking help from health professionals showed that there were significant misconceptions among the participants. These misunderstandings were UI being a normal aging process and usual among women, and no treatment was available. Also, some felt embarrassed when sharing such symptoms with others, as illustrated in Table 7.

**Table 7:** Reasons for not seeking help by gender.

Reasons for not seek help by gender					
Reasons for not seek help		Total 124(100%)	Gender		p-value
			Male 46(%)	Female 78(%)	
	Not a serious problem	49(39.5)	15(32.6)	34(43.6)	0.2
Hoped for spontaneous resolution of UI		25(20.2)	4(8.7)	21(26.9)	0.01
Embarrassed to visit a male or female clinician		13(10.5)	3(6.5)	10(12.8)	0.2
Embarrassed to visit a male clinician		18(14.5)	0(0.0)	18(23.1)	0.001
Feeling that physician would not pay attention		4(100.0)	0(0.0)	4(100.0)	0.1
Believed that UI was a normal among women		35(100.0)	0(0.0)	35(100.0)	0.001
Believed that UI was a normal aging process		53(100.0)	9(17.0)	44(83.0)	0.001
Unaware that treatment was available		17(100.0)	6(35.3)	11(64.7)	0.8

## Discussion

Regarding the ICIQ severity score, we found the participants whose moderate and severe scores were 78 (62.9%) and 32 (25.8%). This ICIQ severity of UI was higher than the respective 30%, and 17% of the 60-year-old women were from the United Kingdom and Ireland [17]. Also, it is higher than the ICIQ moderate 8.8% and severe 5.3% of Mexican older men and women 70 years and older [18].

Our findings showed a significant association between increasing BMI and the severity of ICIQ. This finding is consistent with a Nurses’ Health Study [19] and other studies [8,20].

Other factors such as increasing age, female gender, a high number of vaginal deliveries, and high menopause age showed a higher ICIQ score of severity, but these differences were not statistically significant. Previous studies showed that the risk factors of UI were multiparty, neonatal macrosomia, and prolonged second stage of labor [7,8].

Regarding the associated health conditions (risk factors) versus the ICIQ score of severity of urinary incontinence, our study showed that the participants with hypertension, diabetes, nocturnal diuresis, obesity, arthritis, benign prostatic hypertrophy, fecal incontinence, lung disease, heart disease, stroke, and UTI suffered from a high ICIQ score. The differences were statistically significant in the case of nocturnal diuresis, UTI, and lung diseases. Studies on diabetic patients showed that diabetes mellitus was a significant risk factor for a high ICIQ score of severity [21,22]. Also, a study of males showed that there was an association between urinary incontinence in males and chronic obstructive pulmonary disease (COPD) [23]. Also, a study of Turkish women has found that hypertension, fecal incontinence, history of natural enuresis, and recurrent UTIs have a strong association with UI [24].

In our study, the behavior of seeking help from health professionals among our participants was low. Surprisingly, they sought help from a layperson such as a spouse more than from doctors. The study of Moroccan and Turkish women reported similar low help-seeking for UI [11]. There were multiple explanations for the various misconceptions reported by our participants for not seeking help. First, patients perceived UI as a normal process of aging, consistent with the Netherlands findings [25]. Some individuals do not consider UI to be a serious problem. Furthermore, women hoped for spontaneous resolution. UI patients may feel too embarrassed to consult a clinician of the opposite sex, particularly women; thus, this might further demotivate them from seeking care. They also felt ashamed, so they did not talk about their condition and were socially isolated. This finding is consistent with another study from a similar cultural background population [11,26]. Other reasons for not seeking care are that patients feel that physicians might not pay attention to them, or they are unaware that treatment for UI is available. These findings are consistent with those from studies conducted among Moroccan and Turkish women in the Netherlands [11]. About one in five to one in three urinary-incontinent participants suffered from physical limitation, psychosocial impairment, and unhygienic feelings regarding ablution and prayers. This problem may lead them to isolate themselves from society. In the usual condition, our participants have to conduct ablution before performing their ritual prayers, which occur five times a day, and the urinary dribbling breached their status of purity. Besides the physical, social, and psychological burden of urinary incontinence, some patients may carry themselves an additional burden due to a misunderstanding of their consequences regarding ablution and Prayer. In general, 76.6% of members felt apprehensive and on edge that if they passed urine, they would end up unclean and be denied Prayer until they did the ritual cleansing once more, which is consistent with a previous study [27]. As a result, some people may think that they had to wash more often and repeat ablution whenever they feel some dribbling and experience this as a heavy burden. Our study showed that 83.1% and 66.9% of participants had repeated ablutions and repeated prayers. The previous study reported a similar burden among Muslim Moroccan and Turkish migrant women in the Netherlands [11] and Pakistani women [27]. 

Health professionals should understand their clients’ cultural and religious identities and the problems they encounter in their day-to-day lives. The misconceptions of UI patients should draw attending physicians’ attention in the Muslim community to inquire about this medico-cultural issue. These patients may benefit from the counseling of a Muslim scholar who will explain the religious aspect of coping with permanent urinary incontinence. Addressing the impact of urinary incontinence on the quality of life as a Muslim is crucial. As per Islamic teachings, followers should perform prayers in all circumstances. However, the Islamic Shari’ah is naturally simple and never lays hardship on individuals (The Qu'ran, 22: 78). In instances of hardship and enduring, Shari’ah concessions and licenses consistently emerge. When a Muslim experiences urinary incontinence, and it is continually progressing and does not stop, and there is no available treatment, that Muslims have to hold until the time of the Prayer and afterward begins to expel najasah (ritual impurity) from his body and garments, if conceivable, he/she should set aside clean clothes for Prayer if that is not too difficult. If he/she cannot do that, then he/she is excused. Additionally, he/she can utilize something like a diaper to contain the spread of impurities. Any pollution released from there on will be excusable. At that point, he begins to perform ablution and the Prayer. In extraordinary cases of incontinence, when it is not very easy to follow these steps, one may combine Zhuhr and Asr at the time of either of them. Also, one may combine Maghrib and Isha at the time of either of them [28].

This study suffers from a few limitations. Firstly, it is cross-sectional, depending on the self-report method in two centers only. Thus, the probability of recall bias cannot be excluded. Furthermore, some of the participants, at first, denied having urinary incontinence due to either the misunderstanding of urinary incontinence or the embarrassment that made them withhold the truth while answering the survey. As a consequence, we cannot ascertain the generalizability of our findings. 

## Conclusions

More women than men suffered from various types of urinary incontinence. A large proportion of the participants reported that it had a detrimental effect on their quality of life. Nonetheless, one-third of them did not seek help because of misunderstandings about the disease and its treatment. Some have felt embarrassed, and this stands as an obstacle to seeking medical help.

Based on these findings, we recommended raising awareness of the high prevalence of urinary incontinence to gain the attention and help it deserves. Additionally, physicians should work on improving help-seeking behavior in those with urinary incontinence by overcoming embarrassment. Also, health professionals should be culturally sensitive to their patients' needs and correct their misconceptions.
